# Notes and Comments

**Published:** 1894-08

**Authors:** 


### Notes and Comments.[1]

PATHOLOGY OF THE ALVEOLAR DENTAL MEMBRANE.—Mr. E. Lloyd Williams read a paper on this subject at a recent meeting of the British Dental Association, which is reported in the *Lancet*. He objects to the term "abscess sac," so often applied to an enlarged and inflamed alveolar dental membrane, as being both misleading and unscientific. In the first place, he says, it is not a sac at all, but a solid mass; and, secondly, in the great majority of cases there is no definite abscess to be found. With regard to the cause of inflammation of the root membrane, septic infection is by far the most common, as in nearly all cases it occurs where the pulp is dead and decomposed. According to Mr. Williams, no such thing can take place as a localized periostitis, the whole membrane being invariably affected, though the severity of the attacks may vary in

[1] The assistant editor solicits contributions for this department,—new methods, new remedies and formulas, or any short practical note which may prove of value to the practitioner or student. Address 212 South Fifteenth Street, Philadelphia.

different parts. In the earliest stage of alveolar dental periostitis there is an infiltration of cells into the tissues; later a very loose arrangement of tissue of an adenoid character is seen; and, finally, a large number of areas of inflammation are apparent where the leucocytes have collected and are in sufficient quantity to justify the statement that pus is present; but these foci may so coalesce as to form one large suppurating cavity. Beyond these foci of inflammation a varying thickness of fibrous tissue is found. Micro-organisms, chiefly cocci and rod-shaped bacilli, are found in abundance, more especially in the neighborhood of the blood-vessels, but the essayist failed to discover any in the fibrous tissue or "limiting membrane," as it might be called. With regard to the changes in the contiguous structures, the surface of the bone next the fibrous tissue is, as might be expected, covered with a layer of giant cells, and the cementum, together with the dentine, shows signs of absorption with redisposition, in a great many cases going on synchronously.

---

Plaster of Paris.—The following methods of testing the quality of plaster of Paris are given in the *Charlotte Medical Journal:* First take a small pinch of the powder between the thumb and finger, and gently rub it; if small particles of grit are felt, it indicates that parts of the plaster have already absorbed water, and it is, therefore, unfit for use. The same test may be observed by taking a pinch of the powder again and, placing the fingers under water, rubbing in the same way as before. If, however, in both of these tests no grit is felt, and under water a thin, creamy substance is formed, which is easily rubbed off the fingers, the plaster is in a proper condition for use. Where the plaster has been kept for a long time, or where it is gritty, its condition can be greatly improved. It may be redried by putting it in a metal dish, such as a pie-plate or iron pot, and placing it in an oven of a hot stove or over a gas-jet. As soon as it becomes heated it will be observed that a process identical with boiling water is taking place. When this ebullition has entirely ceased, the powder is freshly kiln-dried. If the method of testing is again resorted to, it will be found that the gritty appearance and feeling will have disappeared in a very large measure, leaving only the fine dry powder ready for use. If there are any lumps remaining, they may be removed by the use of a sieve. From what has been already said, it will need be but a reminder that the plaster of Paris must always be kept in an hermetically sealed jar or in a very dry place.

Asepsis and Antisepsis.—Henry R. Wharton, M.D., contributes to the *Pacific Dental Journal* an exhaustive paper upon the "Theory of Asepsis and Antisepsis in Wound Treatment."

In the introductory of this article, the doctor says the term "asepsis," applied to wounds, implies that the wound is free from those vegetable parasites or micro-organisms whose presence sets up fermentative changes, accompanied by suppuration and constitutional disturbances.

"Antisepsis," on the other hand, has reference to the means employed to bring about the destruction of the micro-organisms which may be present in the wound or upon the instruments, dressings, or hands of the surgeon, and which, if not destroyed or rendered inert, will set up fermentative changes in the wound.

It has long been a well-recognized fact that albuminoid substances, such as dead animal tissue, blood, or blood-serum, will, when exposed to moisture, warmth, and the presence of certain living organisms or fungi, bacteria, and micrococci, develop putrefactive changes; and if these changes take place in the living body, there result certain constitutional disturbances, known as symptomatic, inflammatory, or septic fever.

It was also recognized that these putrefactive changes in albuminoid substances could be avoided by their exposure to heat or cold, or by drying, any of these conditions being sufficient to destroy or arrest the development of the micrococci. The micro-organisms which set up fermentative and putrefactive changes in animal tissue exist in great variety, but those which are of most interest to the surgeon or dentist belong to the "cocci" and "bacilli."

---

Septic Infection from a Bur.—The following note is made from an article by Dr. W. G. Beers, in the *Dominion Dental Journal*, illustrating the danger of careless or dirty dentistry: A young lady in perfect physical health, and whose mouth was as fresh and sweet as a rose in June, was having a lower molar cervical cavity prepared by a dentist, who only used a rickety old engine, but whose burs were so blunt that he had to put on extra pressure to make them cut.

Twice one of the largest burs slipped out of the cavity, tore the gums, and plunged into the mucous folds lying between the tooth and the cheek, revolving there several times before it could be withdrawn. The patient was dismissed after the cavity was filled, without the least attention being paid by the operator to the wound he had inflicted.

About twenty-four hours after the accident the gum in the vicinity became tender and turgid. The face was swollen, the inflammation extending along the inferior maxillary, involving the depressor anguli oris, etc.

The inflammation facilitated the ingress of pathogenic bacteria, which are frequently, if not always, present in the mouth, on the *qui vive* for abrasions and wounds, and the consequences followed. To be brief, I made deep incisions directly into and across the place where the bur had entered with a fine lancet, then syringed fully and freely a four-per-cent. solution of carbolic acid, as hot as the patient could bear it, and packed the wound with absorbent gauze soaked in the solution. The following day I syringed thoroughly with hydrogen peroxide, which was followed by abundant effervescence. The gauze had kept the wound well opened, and the syringing with the peroxide did not, as it might if the orifice were small, distend the tissues and partially close the opening. To accomplish this, the lancing was thoroughly done. The next day I treated the case with pure oil of cinnamon on strips of gauze, followed by hot-water injections. On the eighth day the normal signs returned; the patient in the mean time had syringed every day with a mild antiseptic wash. What at first appearance seemed to prognosticate a dangerous case of septicæmia disappeared under the treatment. The constitutional disturbances were so great that the patient lost eleven pounds in weight, and, though convalescent, is still under the care of the family physician.

